# In Vitro Cell Surface Marker Expression on Mesenchymal Stem Cell Cultures does not Reflect Their Ex Vivo Phenotype

**DOI:** 10.1007/s12015-024-10743-1

**Published:** 2024-06-05

**Authors:** Ye Cao, Anna L. Boss, Scott M. Bolam, Jacob T Munro, Haemish Crawford, Nicola Dalbeth, Raewyn C. Poulsen, Brya G. Matthews

**Affiliations:** 1 Department of Molecular Medicine and Pathology, University of Auckland, Private Bag 92-019, Auckland, 1142 New Zealand; 2 Department of Obstetrics and Gynaecology, University of Auckland, Auckland, New Zealand; 3 Department of Surgery, University of Auckland, Auckland, New Zealand; 4 Starship Childrens Hospital, Auckland, New Zealand; 5 Department of Medicine, University of Auckland, Auckland, New Zealand; 6 Department of Pharmacology, University of Auckland, Auckland, New Zealand

**Keywords:** Mesenchymal stem cells, Periosteum, Progenitor, Prospective isolation, CD146

## Abstract

Cell surface marker expression is one of the criteria for defining human mesenchymal stem or stromal cells (MSC) in vitro. However, it is unclear if expression of markers including CD73 and CD90 reflects the in vivo origin of cultured cells. We evaluated expression of 15 putative MSC markers in primary cultured cells from periosteum and cartilage to determine whether expression of these markers reflects either the differentiation state of cultured cells or the self-renewal of in vivo populations. Cultured cells had universal and consistent expression of various putative stem cell markers including > 95% expression CD73, CD90 and PDPN in both periosteal and cartilage cultures. Altering the culture surface with extracellular matrix coatings had minimal effect on cell surface marker expression. Osteogenic differentiation led to loss of CD106 and CD146 expression, however CD73 and CD90 were retained in > 90% of cells. We sorted freshly isolated periosteal populations capable of CFU-F formation on the basis of CD90 expression in combination with CD34, CD73 and CD26. All primary cultures universally expressed CD73 and CD90 and lacked CD34, irrespective of the expression of these markers ex vivo indicating phenotypic convergence in vitro. We conclude that markers including CD73 and CD90 are acquired in vitro in most ‘mesenchymal’ cells capable of expansion. Overall, we demonstrate that in vitro expression of many cell surface markers in plastic-adherent cultures is unrelated to their expression prior to culture.

**Graphical Abstract:**

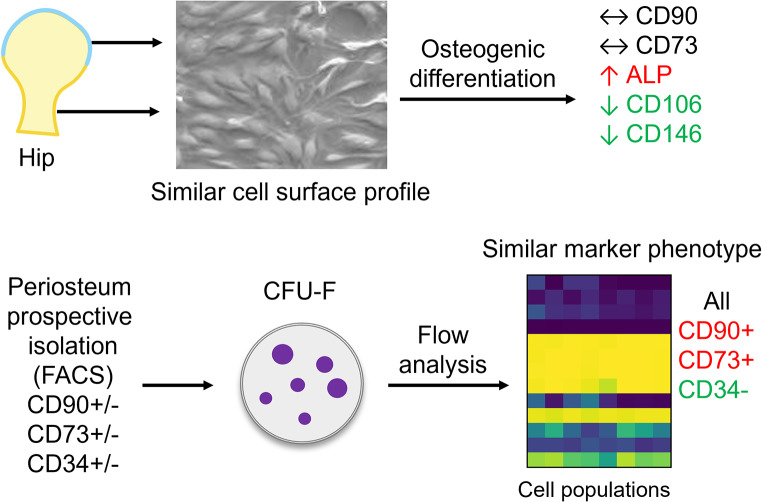

**Supplementary Information:**

The online version contains supplementary material available at 10.1007/s12015-024-10743-1.

## Introduction

The use of mesenchymal stromal cells (MSCs) has been proposed as a therapeutic approach for a huge range of conditions. These cells have properties including extensive expansion potential, multilineage differentiation potential, and the ability to produce various immunomodulatory molecules. MSCs can be isolated from a variety of tissues, although recent studies have confirmed that cell populations from different sources do not attain identical properties [1, 2]. In order to achieve consistency of cell products, the International Society for Cellular Therapy (ISCT) recommends the use of minimal criteria to define in vitro expanded human MSCs [3]. These criteria include adherence to plastic, a cell surface phenotype that lacks several hematopoietic lineage markers and > 95% of the cells expressing CD73, CD90 and CD105, and ability to undergo differentiation to osteoblasts, adipocytes and chondrocytes under permissive conditions in vitro. Despite the attempts by the ISCT to acknowledge that bulk MSC cultures are tissue specific and likely contain a heterogeneous mix of cells including fibroblasts, myofibroblasts and progenitor cells and change reporting practices in the field, they are still widely referred to as mesenchymal stem cells [4, 5]. As such, expression of these cell surface markers is being widely used in the literature to imply that the cells are in an undifferentiated state.

Culturing cells changes their properties and their gene expression. This is partly driven by selection of cells that expand better in culture, but culture-induced changes in marker expression are also common. Culture conditions, including the culture medium employed and the degree of confluence are established to affect cell growth and expression of various markers [6, 7]. There is a longstanding recognition that some markers, particularly CD34, can be lost during the transition to in vitro culture [5, 8, 9]. Likewise, chondrocytes are widely recognized to undergo dedifferentiation to fibroblasts in culture [10]. The culture microenvironment and biomechanical signals likely drives the adoption of a fibroblast-like phenotype in stromal cells from various sources. Nonetheless, there remains an assumption by some in the MSC and tissue engineering fields that in vitro marker expression represents the in vivo cell source from which they were derived. For example, some studies have used the same marker sets identified in vivo to demonstrate in vitro self-renewal of mouse and human skeletal stem cell populations [11, 12], or to propose new markers for prospective isolation of adipose MSCs with specific properties [13, 14]. In the placenta, markers expressed in vitro have been utilized to identify in vivo cell subsets and localization, without proving that the in vivo populations could actually give rise to in vitro cultures with similar properties [15, 16].

Flow cytometry is a powerful single-cell technique enabling simultaneous evaluation of numerous markers and isolation of specific cell phenotypes for downstream functional analyses. The latest spectral instruments enable simultaneous evaluation of 10s of markers. In the current study we have evaluated expression of a panel of 15 cell surface markers that have been proposed to identify mesenchymal stem and progenitor cells in primary cultured skeletal cells. We evaluated whether expression of these markers is influenced by the type of culture coating used, and if their expression changed during in vitro differentiation. We also evaluated if marker expression indicated self-renewal of in vivo populations expressing selected markers following in vitro expansion of various prospectively isolated cells. In this study, we consider marker expression on ex vivo cells that have been isolated directly from human tissue following digestion to indicate their in vivo expression, as these freshly isolated cells have never been cultured. In vitro expression refers to expression in cells that have been cultured on plastic, in this case for a period of at least 6 days.

## Methods

### Collection of Adult Human Skeletal Samples

Collection and use of human tissue was approved by The New Zealand Health and Disability Ethics Committee (NTX/05/06/058/AM15 or 21/CEN/191) and all participants provided written informed consent. Femoral heads were collected from patients (6 males and 5 females, average age 64, range 55–82) undergoing hip arthroplasty for osteoarthritis at Auckland City Hospital or MercyAscot Hospitals, Auckland, New Zealand. Two of these patient samples were included in the cell sorting studies we published recently [17], but the rest are unique to this study. Specimens were kept in sterile saline at 4 °C for no longer than 6 h before dissection.

### Cell Isolation from Skeletal Tissues

Periosteum and cartilage were isolated from femoral heads as described previously [17]. Briefly, the periosteum was scraped off the cortical ring and minced. Macroscopically undamaged articular cartilage was dissected and cut into approximately 2 mm^2^ pieces. Tissues were incubated with 5–10 mL/g tissue of 1 mg/mL collagenase P (Cat: 11-213873001, Sigma-Aldrich) in αMEM 10% fetal bovine serum (FBS), at 37 °C, 100 rpm overnight (< 15 h). Following digestion, cells were filtered through a 70 μm cell strainer (Falcon) and washed with PBS.

### In Vitro Cell Culture and Differentiation

Cells for bulk culture were seeded in αMEM 10% FBS and cultured in a 37 °C humidified incubator with 5% CO_2_. Periosteum cells were seeded at 1.5 × 10^4^ cells/cm^2^ and cartilage at 2 × 10^4^ cells/cm^2^. Half and full media changes were performed on days 4 and 7, respectively. Cells were washed with PBS and detached by StemPro Accutase Cell Dissociation Reagent (Gibco, ThermoFisher Scientific) for passaging once they reached confluence.

For undifferentiated samples and osteogenic differentiation, cells were subcultured in 6-well cell culture plates, at 1.5 × 10^4^ cells/cm^2^, and 2 × 10^4^ cells/cm^2^ for periosteum and cartilage, respectively. At day 2, and day 3 following subculture, when cells from periosteum and cartilage samples reached confluence, 2 wells/sample were collected for flow analysis as undifferentiated samples, and the remainder were changed into osteogenic differentiation media (αMEM 5% FBS, 50 µg/mL ascorbate-2-phosphate (A2P), 5 mM β-glycerophosphate, 10^− 8^M dexamethasone). Osteogenic differentiated cells for flow analysis were collected with 30 min 2 mg/mL collagenase P digestion on day 9 of differentiation. Two wells/sample continued with differentiation until day 21, then were fixed in 10% formalin, and von kossa staining was performed with 1.25% silver nitrate. Cells were imaged using an Olympus IX73 microscope at 4×.

For chondrogenic differentiation, cells were subcultured at 5 × 10^4^ cells per 25 µL spot in 24-well plates (Greiner Bio-one cat # 662,160). After 2 h incubation, 500 µL 10% FBS αMEM was added. The following day, chondrogenic differentiation media (high glucose DMEM, 50 µg/ml A2P, 100 nM dexamethasone, 1× sodium pyruvate, 1× ITS + 1, 40 µg/mL L-proline, and 10 ng/ml TGF-β3) was added, and the cells cultured at 37 °C with 5% oxygen and 5% CO_2_. For flow analysis, cells were detached with Accutase on day 4–5 of differentiation. Two wells/sample continued with differentiation until day 14, alcian blue staining was performed with 1% alcian blue 8 GX in 3% acetic acid, pH 1.0 overnight at room temperature followed by 3% acetic acid (pH 1.0 and pH 2.5) washes. Media changes for both differentiation cultures were performed every 2–3 days.

### Plate Coating Study

This study was performed with either standard 6-well plates (Greiner Bio-one cat # 657,160), or these plates with one of three types of plate coating. Collagen-coating was performed with 0.15 mg/ml rat tail type I collagen (Corning) in 0.02 M acetic acid. Fibronectin coating was performed with 10 µg/ml human fibronectin (Corning). Both were incubated for 1 h at RT then allowed to air dry before use. Geltrex diluted 1:90 with αMEM was added to plates for 1 h at RT then removed immediately before seeding of cells. Primary cells were seeded directly into coated plates at a density of 150,000 cells/well for periosteum and 250,000 cells/well for cartilage. Media changes were performed on day 3 and 5 prior to flow analysis on day 6.

### Flow Cytometry and Cell Sorting

Single cell suspensions of cultured cells were obtained using Accutase, then passed through cell strainers prior to staining. The panel is shown in Supplemental Table 1. This is a simplified version of the spectral panel we previously used in freshly isolated samples [17]. CD24, CD200 and podoplanin (PDPN) were not included in all experiments. Staining and washes were performed in staining medium (SM, 2% FBS, 1 mM EDTA in PBS), and cells were stained in 100 µL antibody cocktail that contained BD Brilliant Stain buffer for 30 min at 4 °C in the dark prior to washing and resuspension. DAPI (50 ng/mL final concentration) was added to each tube prior to acquisition. Analysis was performed on a Cytek Northern Lights instrument with three lasers at the Auckland Cytometry ShaRE.

**Table 1:** Cell surface marker expression (% positive) in the mesenchymal/stromal fraction of freshly isolated or cultured cells from periosteum and cartilage tissue

		Periosteum		Cartilage	
Marker	Gene	Fresh isolated^1^	Cultured^2^	Fresh isolated	Cultured
ALP	*ALPL*	3.9	8.8	13.0	19.0
CD24	*CD24*	1.0	0.2	2.7	9.3
CD26	*DPP4*	21.9	74.2	10.6	7.9
CD34	*CD34*	28.8	4.9	2.2	0.1
CD51	*ITGAV*	22.9	99.0	17.4	56.7
CD73	*NT5E*	34.6	99.4	36.2	99.6
CD90	*THY1*	19.5	96.9	10.0	98.1
CD105	*ENG*	1.4	93.0	10.6	56.0
CD106	*VCAM1*	4.3	41.1	16.9	6.9
CD146	*MCAM*	2.0	98.7	1.9	59.2
CD164	*MUC24*	4.8	94.7	18.7	14.1
CD200	*CD200*	24.3	54.0	10.6	99.6
CD271	*NGFR*	2.1	29.8	6.2	0.6
PDGFRα	*PDGFRA*	2.2	77.3	9.3	98.4
PDPN	*PDPN*	25.4	98.7	54.2	99.5

1. Freshly isolated, *n* = 21, dataset published in [17]

2. Confluent passage 1 cultured cells from different donors, *n* = 3

Cell sorting was performed on freshly isolated periosteal cells as previously described [17]. Briefly, cell pellets underwent red blood cell lysis prior to a final wash. Simple cocktails using the antibodies in Supplemental Table 2 were used for sorting. Staining was performed as described above using 100 µL cocktail volume. Cells were sorted using a BD FACS Aria II, and sorted cells were collected into 1.5 mL sterile tubes containing 500 µL αMEM 20% FBS on ice. Sorted cells were seeded at 300–500 cells/well in 6-well plates and cultured in αMEM 20% FBS at 37 °C with 5% oxygen and 5% CO_2_ for primary CFU-F formation. Half media changes were performed on day 4 and day 7, and colonies were detached with accutase and collected in SM for flow analysis on day 9.

### Data Analysis and Statistics

FCS files were exported and analysed with FCS express 7.18.15 and FlowJo v10.8.1 (BD Biosciences) after unmixing. All the analysis shown is on live, single, hematopoietic and endothelial lineage-negative cells (see serial gating Supplemental Fig. 1). For cultured cells, this means CD45^-^/CD31^-^. Unstained samples from each cell type were used to determine gates for individual markers. Data on graphs are shown as mean ± standard error of the mean. Most studies included *n* = 3 patients. Exact n values and statistical tests performed are detailed in the figure legends.

## Results

### Cell Surface Phenotype Changes in Culture

In preliminary studies, we noted that cultured cells showed much brighter and more consistent marker expression than freshly isolated cells that did not necessarily closely reflect the marker expression in the parent tissue. This is illustrated for periosteum and cartilage cultures in Table 1. Notably, markers including CD73, CD90 and PDPN are almost universally positive in both cell types following culture, while CD34 expression is mostly lost. Given that our previous study demonstrated that CD90^-^ cells in both periosteum and cartilage and CD73^-^ cells in periosteum can form CFU-F in addition to cells positive for both markers [17], we set out to determine how closely this in vitro expression profile reflected the in vivo cells of origin, and the differentiation state of the cultured cells.

### Extracellular Matrix Plate Coating has Minimal Effect on Cell Surface Profile

**Fig. 1:**
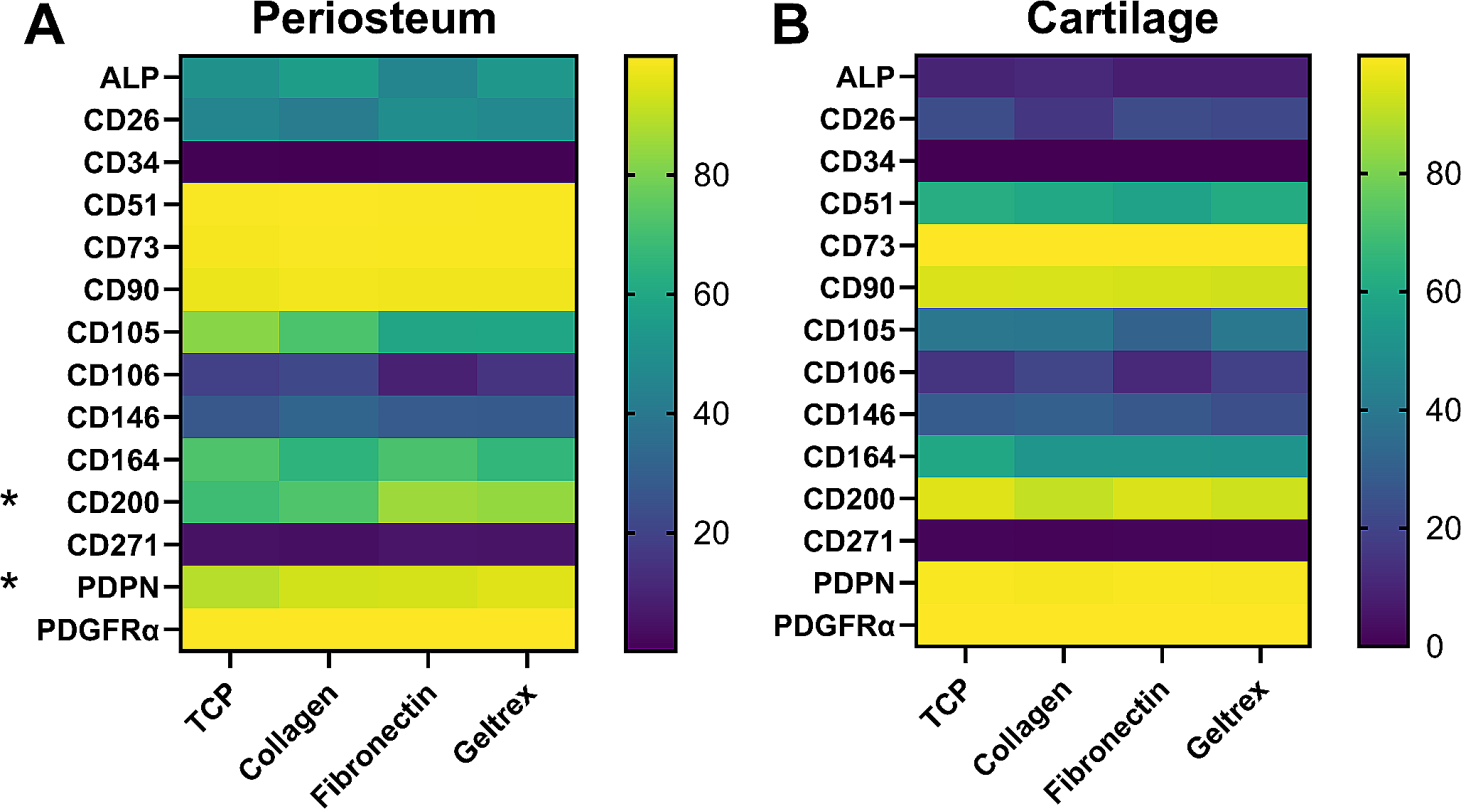
Plate coating minimally affects cell surface marker expression in vitro. The average percentage of CD45^-^CD31^-^ cells expressing the markers indicated at day 6 of primary culture. Cartilage and periosteum were obtained from the same patients, *n* = 3. Different extracellular matrix plate coatings were compared to tissue culture plastic (TCP). Data analyzed by two-way ANOVA with Tukey’s post hoc test. * *p* < 0.05 for comparisons between TCP and at least one of the coatings

One approach to attaining a more in vivo-like environment is precoating plates with extracellular matrix proteins. Fibronectin-coated plates are frequently used to grow chondroprogenitors from cartilage [18, 19]. We coated plates with either type I collagen, fibronectin, or Geltrex (also known as Matrigel) and seeded them directly with freshly isolated cells. The cell surface marker profiles after six days in culture are shown in Fig. 1. While the plate coating did appear to affect attachment and cell morphology (data not shown), it had minimal effects on cell surface marker expression. Notably, there are no significant differences between plate types in cartilage cultures. In periosteum, the proportion of CD200^+^ and PDPN^+^ cells are significantly higher in some or all of the coatings compared to tissue culture plastic controls, however, particularly in the case of PDPN, these represent very small numerical differences (CD200 69% on plastic, 84–85% coated; PDPN 89% on plastic, 93–95% coated). Overall, plate coating does not appear to substantially alter in vitro expression of these cell surface markers.

### CD106 and CD146 are Downregulated during in Vitro Differentiation

**Fig. 2:**
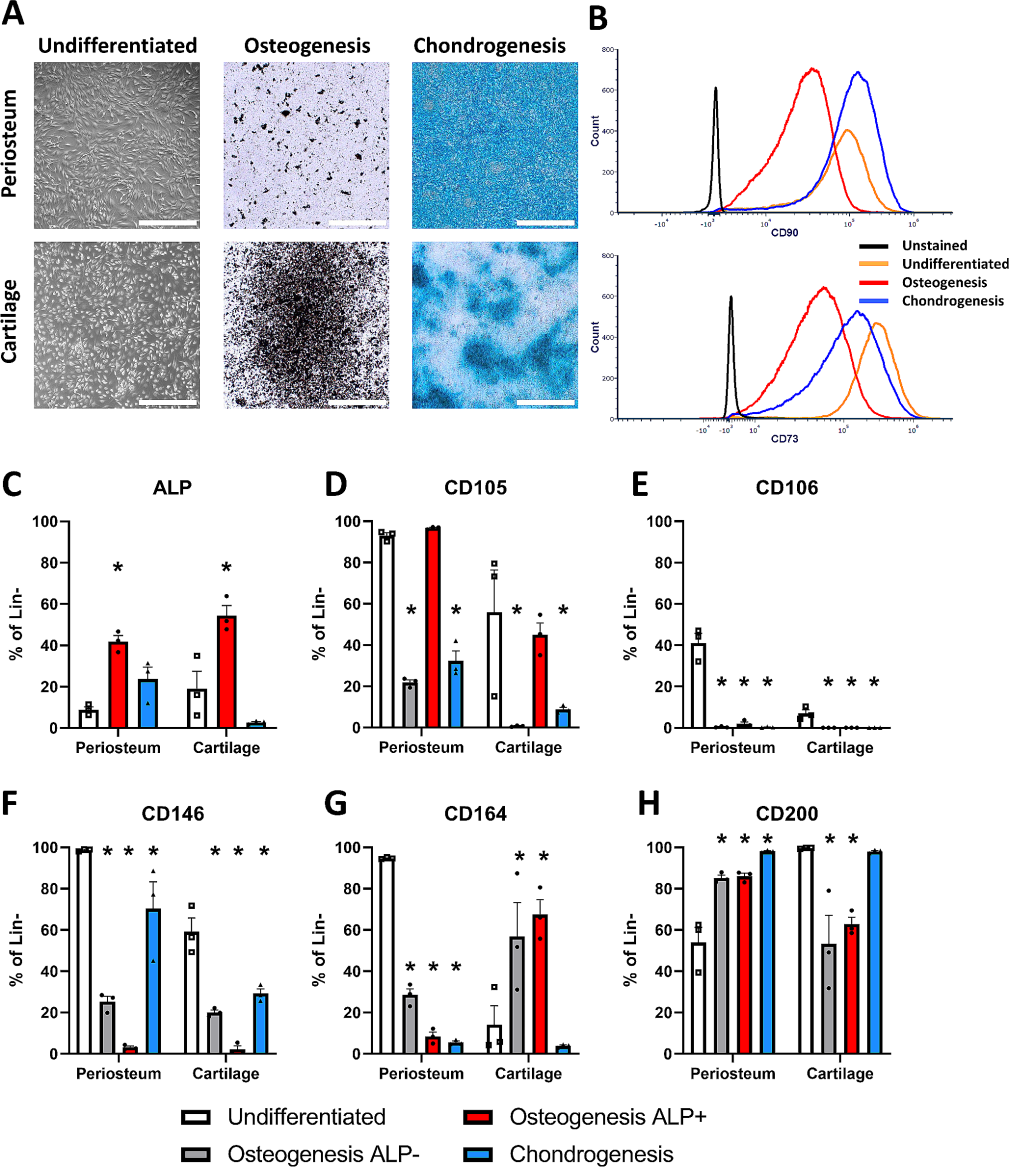
Osteogenic and chondrogenic differentiation alter expression of selected cell surface markers. Passage 1 cells from matched periosteum and cartilage cultures (*n* = 3 patients used for all panels) were cultured under osteogenic and chondrogenic conditions prior to analysis. Undifferentiated cells were analyzed at confluence after culture in basal medium prior to addition of osteogenic medium. Flow analysis was performed on differentiated cells on day 9 (osteogenesis) and day 6 (chondrogenesis). (**A**) Images of a representative culture. Undifferentiated are phase-contrast images at confluence. Osteogenesis shows von Kossa staining after 21 days of differentiation. Chondrogenesis shows alcian blue staining at 14 days of differentiation. Scale bars are 500 μm. (**B**) Representative histograms showing cultured cells are all CD90^+^ and CD73^+^. (**C**-**H**) Selected markers that change following differentiation. One-way ANOVA with Dunnett’s post hoc test performed for each cell type, *p < 0.05 compared to undifferentiated cells

Next, we evaluated whether expression of these markers were regulated during differentiation into osteogenic or chondrogenic lineages. Adipogenic differentiation was not evaluated as mature adipocytes cannot be analyzed by standard flow cytometry techniques, and adipogenesis is not a standard in vivo phenotype for either of these cell sources. Differentiation was induced in passage 1 cells, and surface marker expression was compared to undifferentiated confluent cells prior to initiation of osteogenic differentiation. Flow cytometry of differentiated cultures was performed prior to full mineralization and matrix formation due to the difficulty in generating a single cell suspension from fully differentiated cultures, but organization of cell structures was already evident, and we confirmed that the cultures went on to form mature matrix (Fig. 2A). We included alkaline phosphatase (ALP) as a marker of osteogenic differentiation. Most cells in undifferentiated cultures were ALP^-^, but an ALP^+^ population was evident in both cell types under osteogenic but not chondrogenic conditions suggesting it has utility as a marker of osteogenic commitment (Fig. 2C, Supplemental Fig. 2). When examining how marker expression changed during osteogenesis we split cells into ALP^+^ and ALP^-^ fractions. Several of the markers tested showed little or no change following differentiation, including CD26, CD34 and CD271 (Supplemental Fig. 3). CD73 and CD90 remained expressed in > 90% of cells following differentiation, although notably, the intensity of expression is reduced following osteogenic differentiation (Fig. 2B). CD105 expression decreased in chondrogenic differentiation. Interestingly, in osteogenic differentiation CD105 was decreased in the ALP^-^ fraction while it was retained in the ALP^+^ fraction suggesting CD105 is retained in cells undergoing osteogenic differentiation (Fig. 2D). Similar changes were evident with CD51 (Supplemental Fig. 3). CD106 expression was consistently lost during differentiation (Fig. 2E). CD146 expression was also lost during differentiation, particularly during osteogenesis (Fig. 2F). PDPN expression also slightly reduced during osteogenesis (Supplemental Fig. 3). CD164 and CD200 expression changed during differentiation, but changes during osteogenesis were different in periosteum compared to cartilage cultures (Fig. 2G-H). However, CD164 is mostly absent under chondrogenic conditions while CD200 is universally present. Platelet derived growth factor receptor alpha (PDGFRα) expression decreased during differentiation in cartilage but not periosteal cultures (Supplemental Fig. 3). Overall, our data suggest that none of these markers are expressed in all stem and progenitor cells but inactivated during both osteogenic and chondrogenic differentiation.

### Phenotypic Convergence of Different Cell Populations Occurs with in Vitro Culture

**Fig. 3:**
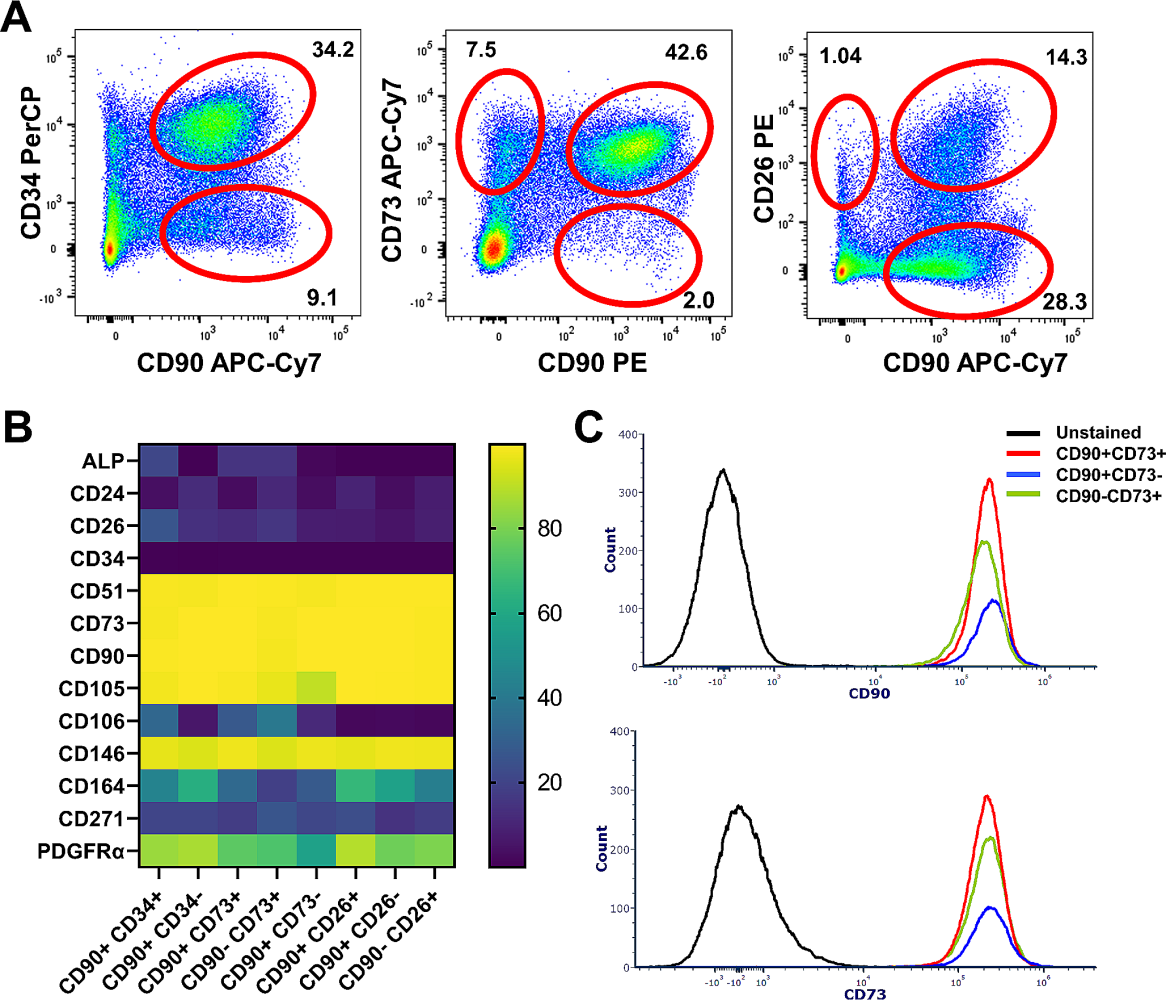
Different prospectively isolated periosteal cell populations express similar markers post culture. (**A**) Representative flow plots showing freshly isolated ex vivo periosteal cell populations sorted for CFU-F formation. All these populations were capable of CFU-F formation (see [17]) to generate enough cells in vitro for flow analysis. (**B**) The percentage of cell surface marker expression within the indicated periosteal cell populations post-culture, *n* = 2–5. Analysis was performed on primary cells, multiple CFU-Fs were pooled. (**C**) Representative histograms showing that CD90^-^ and CD73^-^ cells become CD90^+^ and CD73^+^ post culture

In order to directly evaluate whether marker expression is altered by transition to in vitro culture, we sorted eight periosteal cell populations from freshly isolated tissue that we had previously demonstrated were capable of forming CFU-F based on CD90 expression in combination with CD34, CD73 and CD26 (Fig. 3A) [17]. Following low density culture, primary cells from multiple colonies were pooled for analysis. The overall surface marker profile was very similar for all expanded populations (Fig. 3B). All cells, including the CD90^+^CD34^+^ population are > 99% CD34^-^ post culture. CD26 expression varied from < 1–50% in different samples, but this appeared to be primarily linked to the donor rather than the population that was sorted. CD90 and CD73 were both consistently expressed on > 97% of cells, including in cultures initiated by cells that did not express these markers ex vivo (Fig. 3C). In summary, the in vitro expression of CD26, CD34, CD73 and CD90 does not reflect the in vivo expression of these markers and therefore cannot be used as an indicator of self-renewal.

## Discussion

We set out to determine if in vitro expression of typical cell surface markers in MSC cultures reflected their in vivo origin. We were unable to identify any markers whose expression pattern was retained following prospective isolation. We considered periosteum a useful tissue to undertake this study due to the presence of a high proportion of progenitor cells (1.7% of lineage-negative cells based on CFU-F formation), and the existence of populations with a variety of combinations of frequently used markers capable of clonal expansion ex vivo [17]. We directly demonstrated that CD73 and CD90 expression are upregulated in vitro even in cells where they are initially absent. In vitro upregulation of CD73 has similarly been reported on CD73^-^ placental MSCs after culture [20]. Our data suggests that the same applies to CD51 (unpublished preliminary studies showed CFU-F formation of CD51^-^ cells), CD105, and CD146. In vitro acquisition of CD146 was previously demonstrated in bone marrow [21, 22]. CD26 expression, while variable in vitro, was unrelated to its expression on the population that was sorted. Finally, in line with other studies, we demonstrated loss of CD34 expression in vitro [8, 9]. CD271 is another marker that is often lost or downregulated in culture [20, 21, 23, 24], although that may not be the case in the periosteum given how few cells initially express the marker. Several studies of cartilage cells have demonstrated changes in marker expression following culture, including evidence that CD105 and CD90 expression is acquired in vitro [19, 25]. We did not investigate the mechanism of these changes in marker expression, but they are likely due to the adaptations required to grow in vitro, and acquisition of a more fibroblast-like phenotype [26]. Overall, we have found no evidence from this or previous studies that in vitro expression of cell surface markers that can prospectively identify human stromal stem/progenitor cells reflects the in vivo marker expression of their progenitors, even in cells that have undergone limited expansion.

Stem and progenitor cell markers should ideally be downregulated during differentiation, so we tested the effect of osteogenic and chondrogenic differentiation on marker expression. We do not necessarily expect all cells in culture to differentiate. As expected, ALP was upregulated during osteogenesis and ALP^+^ and ALP^−/lo^ populations were clearly present after osteogenic induction. The marker that showed the most robust downregulation during differentiation was CD106, in line with previous studies in bone marrow-derived cultures and expression data in the human primary cell atlas on BioGPS [13, 27, 28]. However, CD106 was initially only expressed in a subset of undifferentiated cells, including in 1–40% of cells in clonal cultures which should contain a larger proportion of stem/progenitors than bulk cultures, suggesting it may only be expressed in a subset of stem and progenitor cells initially. CD146 was also consistently downregulated during osteogenic differentiation, and to a lesser extent during chondrogenic differentiation. This is consistent with data from bone marrow and adipose MSC differentiation, although notably it is strongly upregulated during adipogenesis [13, 14]. Our data indicate that the typical MSC markers CD73 and CD90 are reduced during osteogenesis, but not to an extent that would be useful for evaluating differentiation status, consistent with data in adipose MSCs [14]. CD105 expression was lost in cultures undergoing differentiation in periosteal and cartilage cultures in addition to bone marrow and adipose MSCs [13, 14]. Surprisingly, it was lost specifically in ALP- cells, which we expect would include mainly less differentiated cells in the culture. A flow-based assay to evaluate differentiation status of cultures could provide rapid but more nuanced data on culture status than histochemical staining, or possibly even bulk gene expression, but our data indicates that multiple markers would be needed with further validation to understand the identity and ongoing expansion and differentiation potential of different cell subsets.

In vitro expansion undoubtedly changes cell characteristics, including their size and gene expression. However, expanded cells retain tissue-specific gene expression and restricted differentiation potential when suitably robust experimental conditions are employed [1]. Nonetheless, culturing on plastic appears to encourage conversion of cells to a fibroblastic phenotype and phenotypic convergence of marker expression [26]. Designing culture conditions that are more in vivo-like is one approach to maintaining cell phenotypes. However, our results demonstrate that coating tissue culture plastic with various matrix proteins did not substantially change cell surface marker profiles to promote retention of ex vivo phenotypes. This aligns with our previous results in rat osteoblast cultures that demonstrated collagen-coated plates did not alter osteogenic gene expression, but culturing in three-dimensional gels dramatically increased osteogenic gene expression [29]. Some investigators have used alternative culture approaches such as growth as mesenspheres to test expansion and self-renewal capacity of putative mesenchymal stem cell populations [30–32]. Mesensphere expansion promoted in vivo self-renewal compared to CFU-F-based expansion [32]. However, mesensphere cultures mostly lost expression of CD105 and CD146 in one study, suggesting that changing cell surface marker expression may remain a feature of this system [31]. It is difficult to determine if mesensphere cultures truly represent a more robust method than culture on plastic to expand stem cell populations without more systematic studies. Maintaining a more in vivo-like phenotype while expanding stem and progenitor cells is probably a useful approach when generating cells for transplantation where the goal is long-term engraftment. Data from mouse studies suggests that successful intramarrow engraftment is possible with freshly isolated cells, although we are not aware of studies that have evaluated engraftment over months to years [33]. Conversely, cultured cells only engraft locally following irradiation [34]. Therefore, future studies should focus on alternative approaches to expand skeletal stem and progenitor cells while retaining their phenotype ex vivo.

We included cultures from cartilage as a comparison to periosteum in the bulk culture studies. Cartilage is not typically used to isolate MSCs, and does not contain pericytes, so cultures derived from cartilage using this type of protocol are often termed chondrocytes. However, there are many reports describing progenitor populations in articular cartilage that may be the main source of cells growing in these cultures [19, 35, 36]. Dedifferentiation to a more proliferative fibroblastic phenotype is also a recognized feature of chondrocyte cultures, and cells show cell surface marker expression that fit MSC criteria [10, 37]. Prior to culture, cartilage cells have remarkably abundant expression of many proposed stem cell markers, and notable differences to periosteum [17]. Nonetheless, we found that cartilage cultures have a similar, but not identical, cell surface marker profile to periosteum post culture. In vitro selection for cartilage progenitors often involves selection based on preferential binding to fibronectin [36]. Fibronectin coating did not change the final marker profile in our cartilage cultures, although notably we did not limit the time allowed for attachment like the published protocols. Cells from adult cartilage and periosteum are both capable of differentiating into chondrocytes and osteoblasts in vivo, even though the type of chondrocytes are different, and osteogenic differentiation in articular cartilage is a pathological process. Nonetheless, we considered these physiologically plausible differentiation fates for these cells. In most cases, the differentiation-related changes in marker expression were similar in both cell types, even when baseline expression of markers varied.

Heterogeneity of various cell types in vitro and in vivo has been documented for decades [38]. Recent advances in single cell technologies and widespread adoption of single-cell RNAseq has revealed a great deal of cellular heterogeneity in the marrow stroma, and in mice helped to clarify previously unappreciated roles of stromal subsets that help to maintain a bone marrow cavity, for example [39, 40]. RNA data can predict cell surface protein expression most of the time [41], providing an approach to identify novel in vivo cell subsets for further investigation. Fewer datasets are available from human bones, but these also demonstrate heterogeneity of populations both ex vivo and in vitro that are not revealed by analyzing a small number of cell surface markers [42–44]. These technologies, which can include antibody-based detection of cell surface markers in parallel with RNA analysis, provide a new platform for discovery of tissue-resident populations that are of interest for prospective isolation. However, with human cells, designing appropriate assays to evaluate their function remains a challenge.

A limitation of this study is the lengthy tissue digestion prior to determining ex vivo marker expression. Tissue digestion is critical to generate suitable samples for flow cytometry and cell sorting. Notably, heterogeneous expression is evident ex vivo for all the markers included in this study suggesting they are not universally gained or lost during the process. We have also previously demonstrated the presence of CD90^+^CD34^+^ and CD90^+^CD73^+^ cells in the periosteum by immunostaining indicating these populations exist in vivo [17]. Differentiation studies were conducted at only one time point, and this was relatively early in the process prior to full matrix assembly. This timing was chosen firstly to represent earlier stages of differentiation that may begin spontaneously in a confluent culture, but also due to practical difficulties generating suitable samples for flow cytometry from fully differentiated cultures.

In conclusion, our data suggest that some markers are expressed in vitro in most ‘mesenchymal’ cells capable of expansion regardless of the tissue of origin, cell subset of origin, or seeding density. CD73 and CD90 in particular were universally present, and barely impacted by differentiation. These markers appear to be upregulated by in vitro culture and their presence is unrelated to whether they were expressed in vivo. CD105 was also frequently abundant, but was not as universally expressed in cartilage cultures. This near-universal expression makes the utility of evaluating these markers routinely in cultures questionable as an approach to demonstrate consistent cell phenotype between studies. Overall, we demonstrate that in vitro expression of cell surface markers in plastic-adherent cultures is generally unrelated to expression in the in vivo cell of origin, and that CD146 and CD106 are candidates for markers that select for undifferentiated cells within the culture.

### Electronic Supplementary Material

Below is the link to the electronic supplementary material.

Supplementary Material 1

## Data Availability

Data are available from the corresponding author upon reasonable request.
