# Supplementary material for: In Vitro Cell Surface Marker Expression on Mesenchymal Stem Cell Cultures does not Reflect Their Ex Vivo Phenotype
Source: Stem Cell Rev Rep. 2024 Jun 5;20(6):1656–66. doi: 10.1007/s12015-024-10743-1 (PMC11319515; doi:10.1007/s12015-024-10743-1)
Supplement: Supplementary file 1 — Supplementary Material 1 [file 12015_2024_10743_MOESM1_ESM.pdf]

Supplemental Table 1. Spectral flow cytometry panel for cultured cells

Antigen	Gene	Fluorophore	Clone	Cat#	Manufacturer	Dose[1]
ALP	*ALPL*	BV650	B4-78	742712	BD optibulid	0.3
CD24	*CD24*	BV711	ML5	311135	Biolegend	0.3
CD26	*DPP4*	PE/Cy5	BA5b	302708	Biolegend	0.075
CD34	*CD34*	PerCP	581	343520	Biolegend	1.25
CD51/CD61	*ITGAV/ ITGB3*	APC	23C6	304415	Biolegend	0.3
CD73	*NT5E*	BV785	AD2	344028	Biolegend	0.15
CD90	*THY1*	Alexa Fluor 700	5E10	328120	Biolegend	0.3
CD105	*ENG*	BV750	266	747107	BD optibulid	0.3
CD106	*VCAM1*	BV421	STA	305815	Biolegend	0.3
CD146	*MCAM*	BV605	P1H12	361024	Biolegend	0.15
CD164	*MUC24*	PE	67D2	130-108-104	Miltenyi Biotec	0.5
CD200	*CD200*	APC Fire™ 750	OX-104	329224	Biolegend	0.3
CD271	*NGFR*	PE/CF594	C40-1457	563452	BD Biosciences	0.3
PDGFRα	*PDGFRA*	PE/Cy7	16A1	323507	Biolegend	0.3
PDPN	*PDPN*	PerCP-eFluor 710	NZ-1.3	46-9381-42	eBioscience	1.25
**Lineage markers**						
CD31	*PECAM1*	BV480™	WM59	566195	BD Biosciences	0.3
CD45	*PTPRC*	Krome Orange	J33	B36294	Beckman Coulter	0.3

1. Antibody dose in μL/tube.

Supplemental Table 2. Additional antibodies for cell sorting

Antigen	Conjugate	Clone	Cat #	Manufacturer	Dose (μL/100μL)
CD26	PE	BA5b	302706	Biolegend	0.15
CD73	APC-Cy7	AD2	344021	Biolegend	2.5
CD90	APC-Cy7	5E10	328131	Biolegend	1
CD90	PE	5E10	561970	BD Biosciences	0.25
**Lineage markers**					
CD31	FITC	WM59	303104	Biolegend	2.5
CD45	FITC	HI30	555482	BD Biosciences	10
CD235a	FITC	HI264	349104	Biolegend	2.5

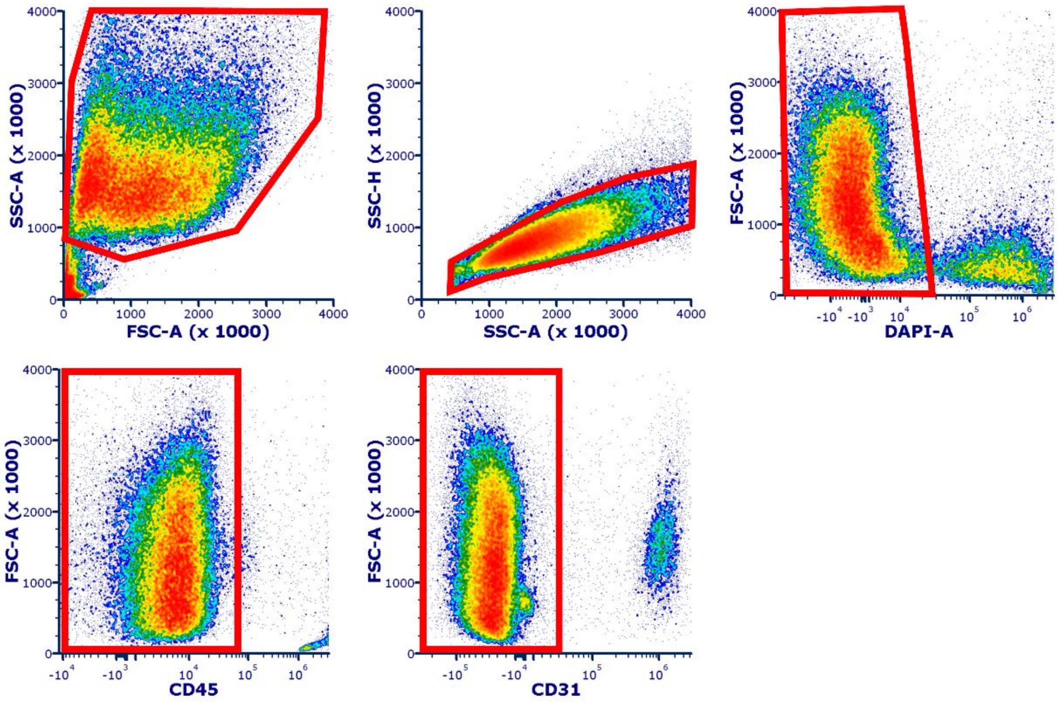

Supplemental Figure 1: Gating strategy to obtain Lin- populations post culture

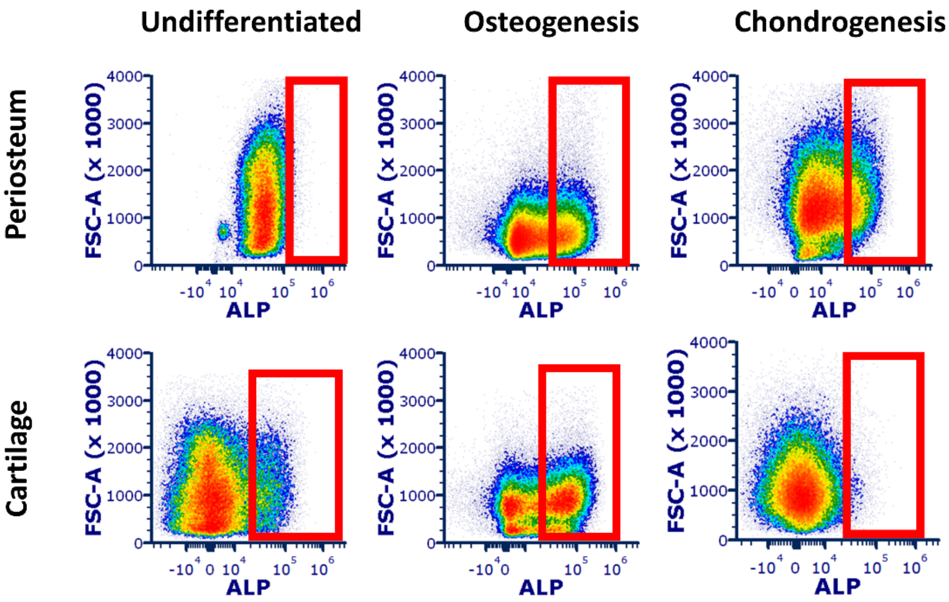

Supplemental Figure 2: ALP expression and gating strategy of ALP populations post culture and differentiation

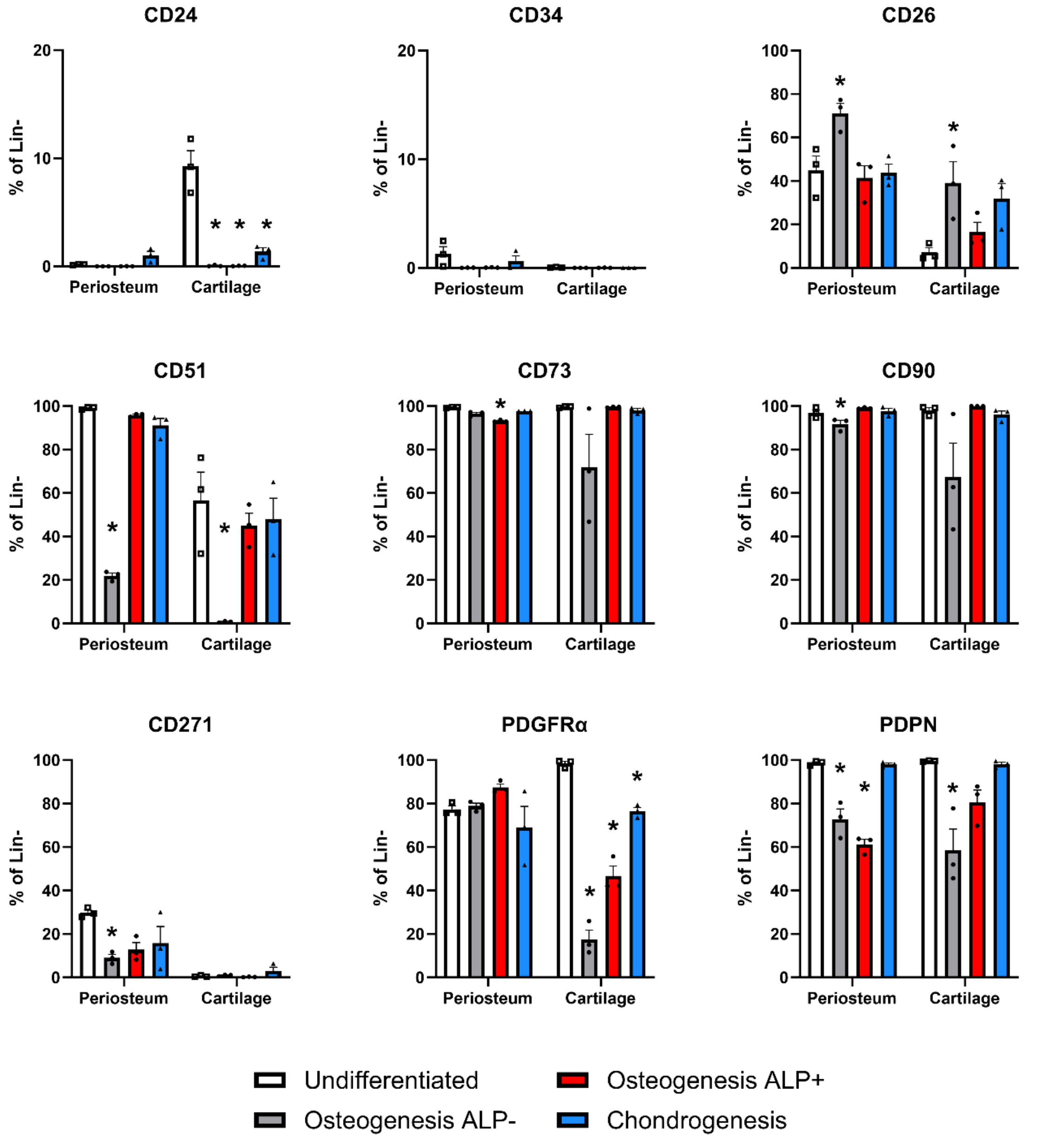

Supplemental Figure 3: Expression of markers that show limited or less consistent changes in expression following in vitro differentiation.

Extended data for Figure 2. Passage 1 cells from matched periosteum and cartilage cultures (n=3 patients used for all panels) were cultured under osteogenic and chondrogenic conditions prior to analysis. Undifferentiated cells were analyzed at confluence after culture in basal medium prior to addition of osteogenic medium. Flow analysis was performed on differentiated cells on day 9 (osteogenesis) and day 6 (chondrogenesis). One-way ANOVA with Dunnett's post hoc test performed for each cell type, *p<0.05 and at least 5% change in marker frequency compared to undifferentiated cells.
